# CFD-DEM Evaluation of the Clustering Behavior in a Riser—the Effect of the Drag Force Model

**DOI:** 10.1021/acs.iecr.3c00853

**Published:** 2023-05-30

**Authors:** Juan Ramírez, Martijn de Munck, Zhitao Liu, David Raphael Rieder, Maike Baltussen, Kay Buist, Johannes A. M. Hans Kuipers

**Affiliations:** Multiphase Reactors Group, Department of Chemical Engineering & Chemistry, Eindhoven University of Technology, Eindhoven, 5600 MB, The Netherlands

## Abstract

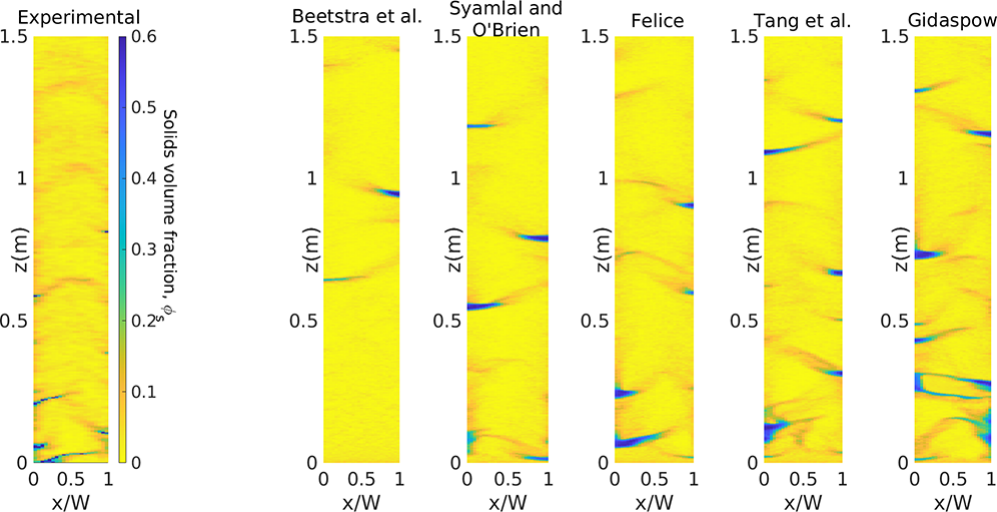

Riser reactors are frequently applied in catalytic processes involving rapid catalyst deactivation. Typically heterogeneous flow structures prevail because of the clustering of particles, which impacts the quality of the gas–solid contact. This phenomenon results as a competition between fluid–particle interaction (i.e., drag) and particle–particle interaction (i.e., collisions). In this study, five drag force correlations were used in a combined computational fluid dynamics–discrete element method Immersed Boundary Model to predict the clustering. The simulation results were compared with experimental data obtained from a pseudo-2D riser in the fast fluidization regime. The clusters were detected on the basis of a core–wake approach using constant thresholds. Although good predictions for the global (solids volume fraction and mass flux) variables and cluster (spatial distribution, size, and number of clusters) variables were obtained with two of the approaches in most of the simulations, all the correlations show significant deviations in the onset of a pneumatic transport regime. However, the correlations of Felice (Int. J. Multiphase Flow1994, 20, 153−159) and Tang et al. [AIChE J.2015, 61 ( (2), ), 688−698] show the closest correspondence for the time-averaged quantities and the clustering behavior in the fast fluidization regime.

## 1 Introduction

Riser reactors find a widespread application in catalytic processes featuring relatively fast deactivation of the catalyst. Despite the geometrical simplicity of this kind of system, the gas–solid flow structure is quite complex because the spatial solids distribution is rarely uniform. One of the consequences of such heterogeneity is the formation of clusters, which are normally defined as regions with a high solids volume fraction.^2,11,25^ Different authors have presented the drawbacks of such clusters with respect to riser performance, such as increased back-mixing and segregation near the wall due to the larger flow resistance in these dense structures, as well as less efficient gas–solid interaction and interphase mass transport.^8,9,14,20,37^

Cluster formation is a particle-scale phenomenon promoted by the collisions of hundreds or thousands of particles and is, therefore, well-suited to be analyzed with a computational fluid dynamics–discrete element method (CFD-DEM) model. In CFD-DEM, each particle is handled individually using Newton’s second law of motion, while the gas phase is treated as a continuous phase by solving the Navier–Stokes and continuity equations. Different authors have evaluated the effect of several system characteristics on the properties of the clusters, such as operational conditions,^18,25,27,32^ collision parameters,^11,25,32^ lift force,^11^ inlet and/or outlet configurations,^18^ and equipment dimensions.^11,27^ Apart from the collision dynamics, the interphase momentum exchange also affects the formation of clusters and the overall operation of the riser. One of the most notable gas–particle interactions is the drag, of which many formulations have been numerically tested.^19,21,32,33,39^ One of the challenges in selecting an appropriate drag model is that the magnitude of the predicted drag force among the different drag closures can vary significantly depending on the values of local gas fraction, ε_*g*_, and particle Reynolds number, *Re*. This is particularly important in a riser because dilute regions (with ε_*g*_ ≈ 1.0) and cluster regions (with ε_*g*_ values as low as 0.38) coexist. Additionally, the strong dynamics in a riser lead to a wide range of possible particle Reynolds numbers with values as high as 500–600. Because of the wide range of *Re* and ε_*g*_ values, the current comparisons of drag correlations in CFD-DEM modeling have only limited applicability because they are generally performed for bubbling fluidized beds.^19,33^

Some of the previous studies addressing the effect of the drag force formulation in risers adopted a Eulerian–Eulerian formulation, also known as two fluid model (TFM).^21,31,39^ However, the solids interactions in TFM are represented in the context of continuum descriptions, e.g., the solid stress and solid viscosity. Generally, TFM shows less predictive capabilities because of the large uncertainties regarding the used closures. In addition, it is generally accepted that the incorporation of realistic and detailed models for particle–particle and particle–wall interactions is more straightforward in the CFD-DEM approach. To the best of the authors’ knowledge, only two studies have addressed the effect of the drag force model on the riser hydrodynamics using CFD-DEM. Wang et al.^32^ compared the formulations of Gidaspow^7^ and Beetstra et al.;^1^ however, this research shows that these two correlations produce results that deviate significantly from the experimental data, e.g., the observed clustering behavior. Xu et al.^38^ broadened the spectrum by including two more formulations (Wen and Yu^36^ and Hill et al.^10^). However, the correlation of Wen and Yu is obtained for homogeneously dispersed systems, and the correlation of Hill et al. is only valid for Reynolds values below 100. The relevance of these correlations in a riser is, thus, limited because dense clusters structures and a relatively high Reynolds number are typically expected. This paper aims to understand the effect of drag force correlations in riser systems by comparing five of the most commonly used formulations applicable for a wide range of local gas fractions and Reynolds numbers of a riser unit. These formulations can be divided into two categories on the basis of the approach followed in their formulation: the approaches of Gidaspow,^7^ Syamlal and O’Brien,^24^ and Felice^6^ belong to the category of semiempirical models correlated on the basis of experimental data, while Beetstra et al.^1^ and Tang et al.^26^ based their correlations on particle-resolved numerical simulations of flow through different arrays of randomly placed particles, using a lattice Boltzmann approach and an immersed boundary method (IBM), respectively. A third category of drag correlations corresponds to those deducted from an energy minimization multiscale (EMMS) approach.^34^ Despite the successful implementation in riser systems,^15,17^ this approach was not considered in this study because this approach is mostly used in dilute systems where the local structures are not captured by the representation of the averaged solids fraction.

In the next sections, first the CFD-DEM model is explained. Subsequently, the drag force models and the clustering detection method will be described. In the results section, the relative difference between the predictions of global variables, such as the solids holdup and solids flux, are presented. Afterward these differences are explained by relating the results to the normalized drag force obtained for the different approaches for the averaged conditions in the riser. Finally, the different implementations of the drag force are assessed on the basis of the predictions in the clustering behavior, which are compared with the experiments in a pseudo 2D riser.^29,30^ Although this work studies the same system as Varas et al.^29^ and Mu et al.,^18^ the current study focuses on the drag formulations used in the simulations, which neither of them discussed.

## 2 Numerical Methods

### 2.1 CFD-DEM

The CFD-DEM representation used in this research, developed simultaneously by Tsuji et al.^27^ and Hoomans et al.,^12^ considers the gas in the riser as a continuous phase. The volume-averaged Navier–Stokes formulation and the continuity equation are used to describe the gas phase hydrodynamics (eqs 1 and 2, respectively).
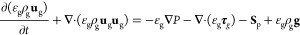
1
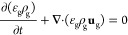
2

Where **S**_p_ represents the source term because of the momentum transfer between the gas and solid particles:
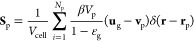
3

The regularized Dirac delta function δ(**r**–**r**_p_) maps the momentum change of each of the *N*_p_ Lagrangian particles to the relevant Eulerian velocity nodes. This same regularized function also maps the gas phase properties from the Eulerian grid to the particles position, thereby enabling the evaluation of the drag force.

The particle motion is described using Newton’s second law of motion:
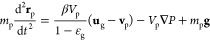
4
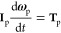
5Equation 4 lacks a force contribution because of collisions with other particles and walls. In the implemented hard sphere approach, these forces are considered to be impulsive and instantaneous, i.e., the duration of that contact is significantly shorter than any of the finite forces on the right side of eq 4. Therefore, the effect of the collisions on the linear and angular momentum can be computed independently at the moment the collision is detected. The exchange of momentum is done using algebraic equations that guarantee conservation of linear and angular momentum before and after the collision (the interested reader is referred to refs (3) and (5)). In this approach, the interactions between particles are handled as a consecutive series of binary collisions. This results in a fundamental problem since multiple contacts should happen simultaneously in a riser, specifically in dense particle regions, such as clusters. Nevertheless, good representation of the solids distribution has been achieved using this approach in a CFD-DEM representation of risers.^16^

### 2.2 Incorporation of the Riser Geometry

As the geometry of the riser does not conform to the 3D Cartesian grid, the second-order implicit immersed boundary method (IBM) of Deen et al.^4^ is implemented and coupled with the DEM.

In this method, the discretized Navier–Stokes equations are adjusted if one of the neighboring cells resides inside the solid geometry. Using a second-order polynomial representation, the velocity component inside the solid is extrapolated on the basis of the no-slip boundary condition at the bend. This results in an adjustment of the linear set of equations to solve the Navier–Stokes equation, while there are no adjustments required to solve the continuity equation. In addition, collision rules are utilized for the interaction with the IBM boundaries. Based on the treatment of impermeable domain boundaries, particles crossing IBM boundaries are subjected to general collision rules.

### 2.3 Drag Force Models

In a CFD-DEM simulation of a riser, the drag force **F**_d_ (first term in the right-hand side of eq 4), accounts for the momentum exchange between the gas and the particles. Two different classes of drag force correlations are considered in this work: the semiempirical correlations and the correlations obtained from particle-resolved numerical simulations. Table 1 provides an overview of the drag correlations used in this work. To compare these different drag force formulations, one key feature to evaluate is the magnitude of the drag force predicted under different conditions. Often, the interphase momentum transfer coefficient β presented in eqs 3 and 4 is used as a basis of comparison. Another option is to use a normalized version of the drag force obtained when **F**_d_ is divided by the Stokes–Einstein relation, which is the exact result for the drag force around a single particle in the low Reynolds number limit:
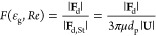
6where **U** is the superficial slip velocity, defined as ε_g_(**u**_g_ – **v**_p_).

**Table 1:** Summary of Drag Force Models Implemented in CFD-DEM Model

approach	origin	equation for *F*(ε_*g*_, *Re*)
Gidaspow^7^	semiempirical	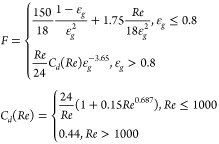
Beetstra et al.^1^	particle-resolved simulations	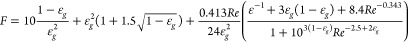
Syamlal and O’Brien^24^^a^	semiempirical	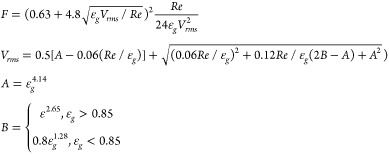
Felice^6^	semiempirical	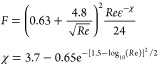
Tang et al.^26^	particle-resolved simulations	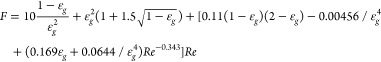

a Syamlal and O’Brien^23^ recommend to calibrate the model with the minimum fluidization velocity. Although this calibration was not performed in this study, the parameters in the model were used from Syamlal and O’Brien^22^ because very similar particles were used in this study.

In this work, we choose to compare the drag formulations using the normalized force since it results in a function of the local porosity ε_g_ and the particle Reynolds number .

**Figure 1:**
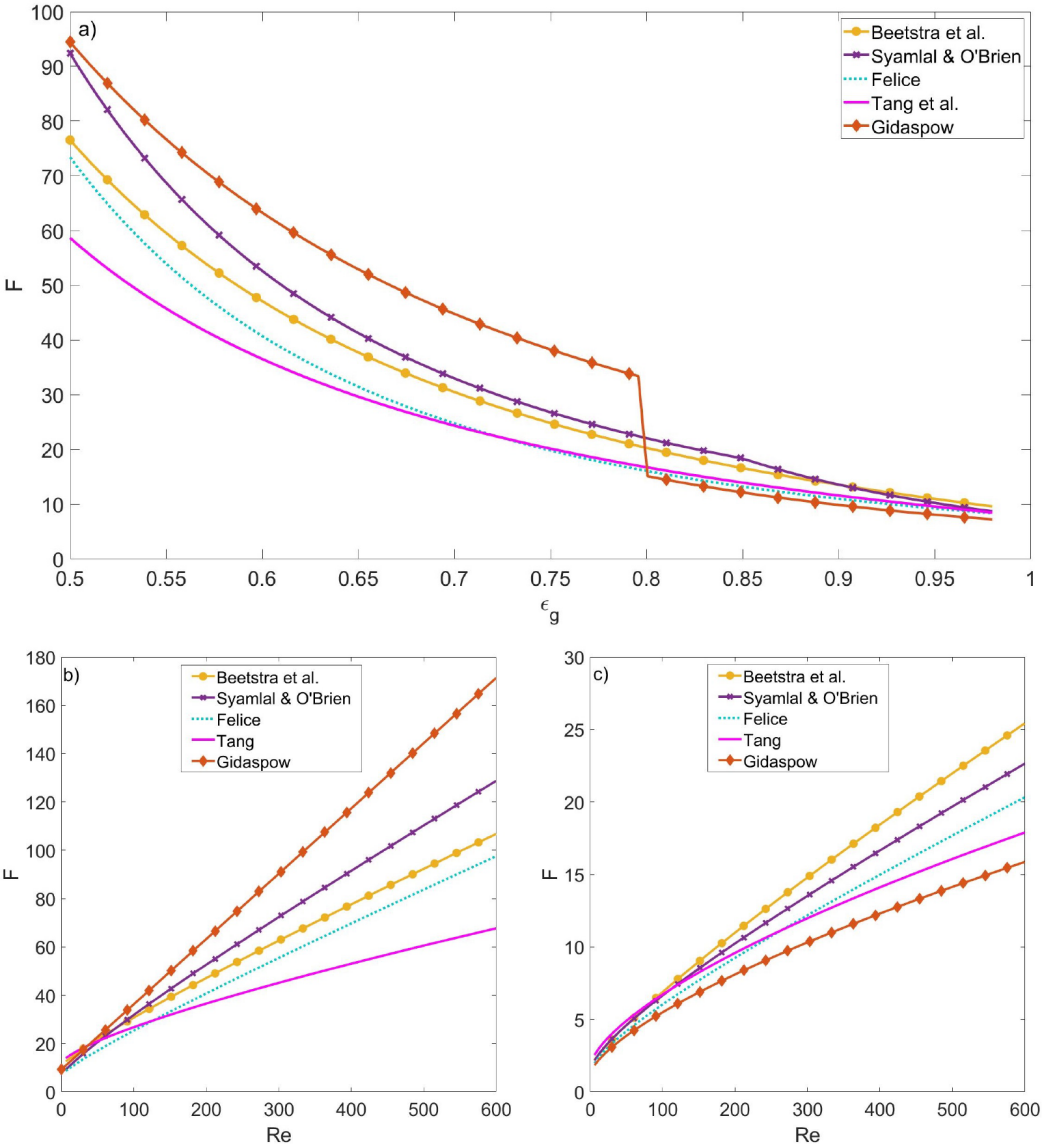
Normalized drag force as a function of (a) the porosity (*Re* = 200) and the Reynolds number, (b) dense condition (ε_g_ = 0.6) and (c) dilute condition (ε_g_ = 0.95).

One key challenge for any of the formulations in Table 1 is the wide range of porosity and particle Reynolds number values that can be encountered in a riser system. Figure 1 shows the behavior of the drag force equations as a function of the porosity (Figure 1a) and the Reynolds number (Figure 1b,c). Although all equations show a decrease of the drag force with increasing porosity (Figure 1a), the differences between the models depend largely on the local porosity and particle Reynolds number. As both dilute and dense regions occur in a riser, an assessment of the behavior of the drag force models at both dense regions (ε_g_ = 0.6, Figure 1b) and dilute regions (ε_g_ = 0.95, Figure 1c) can provide more insight. Comparison of Figure 1 panels (b) and (c) show there are large differences between the relative behavior in the two regimes, which confirms the difficulties in choosing the correct drag model for risers.

### 2.4 Simulation Conditions

**Figure 2:**
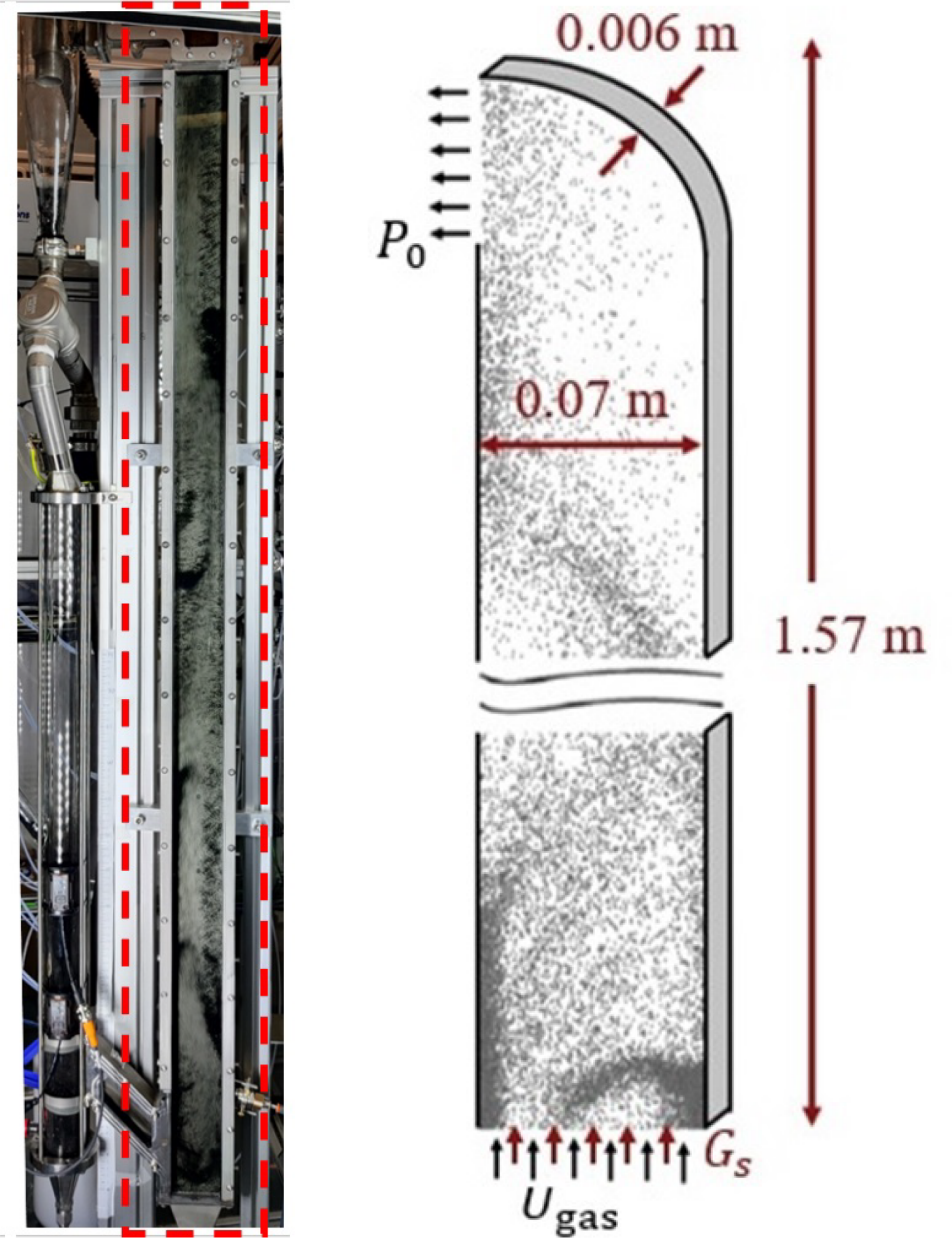
Schematic representation of the riser used in this study.

The simulations are based on the experimental results of Varas et al.^29,30^ A picture and schematic representation with the dimensions of the pseudo-2D riser are shown in Figure 2. The particles that leave the riser at the top enter a cyclone where they are separated from the gas and are directed to the downcomer, which is also shown in Figure 2. This downcomer acts as a storage vessel to keep most of the particle inventory, which is slowly injected back into the riser through a dosage slit. This feeding channel has dimensions of 0.17 × 0.04 × 0.006 m and it is located 0.07 m above the bottom of the riser. The channel is inclined at an angle of 45° and it maintains a feeding rate such that the riser operates at a solids flux of approximately 32 kg/m^2^s.

The exact velocity of the injected particles through the channel is difficult to measure. Therefore, the new particles in the simulations are injected in a narrow section in the bottom of the riser with a low velocity of {0.0, 0.0, 0.1} m/s. The effect on the global predictions of the riser of selecting this approach over a more realistic lateral feeding of the particles has been found negligible.^18^ In the case of overlap of an injected particle with an already present particle in the simulation, the model keeps trying to place new particles without any overlapping maximally 100 times. When unsuccessful, the simulation proceeds without particle injection until the next time step.

For the gas phase, a no-slip boundary condition was defined at the elbow (using IBM), as well as at the top, front, back, and right walls. The left wall (Figure 2) is a combination of a wall (no-slip) in the bottom part and an outflow condition for the final 7 cm, which enforces the outlet pressure to 1 atm. At the bottom of the domain, the inflow was set with *U*_g,in_ = {5.55, 5.95, 6.35, 6.74} m/s. These velocities were considered to study the riser flow along the fast fluidization regime until the onset of pneumatic transport. Table 2 provides an overview of all the parameters used in our simulations. Particle information corresponds to the glass beads used during the experiments. It should be noted that a grid resolution was performed according to Varas et al.,^29^ and the current resolution has proven to be sufficient to obtain accurate results.

**Table 2:** Simulation Settings

*L* (m)	0.07	ρ_p_ (kg/m^3^)	2500
*H* (m)	0.006	*d*_p_ = (mm)	0.85
*D* (m)	1.54	*Δt*_flow_ (s)	5 × 10^–5^
*Δx* (m)	2.5 × 10^–3^	*Δt*_DEM_ (s)	5 × 10^–6^
*Δy* (m)	1.25 × 10^–3^	*e*_*n*_p–p__	0.96
*Δz* (m)	2.5 × 10^–3^	*e*_*n*_p–w__	0.86
*G*_s_ (kg/m^2^s)	32.0	μ_*fr*_p–p__ = μ_fr_p–w__	0.15
*U*_g,in_ (m/s)	5.55, 5.95, 6.35, 6.74	*e*_*t*_p–p__ = *e*_*t*_p–w__	0.33

Each simulation started with an empty column that was gradually filled. Therefore, simulations were performed for 20 s: the first 10 s were used to achieve a pseudosteady state in the riser and the next 10 s were to obtain the desired results. The flow fields and void fraction profiles were obtained every 0.05 s, which resulted in 2000 profiles for all 20 simulations (four gas velocities and five drag force correlations).

The CFD-DEM model used in this work is the in-house code Foxberry.^13^ All simulations were performed on a single thread of an AMD Ryzen Threadripper 2950X processing unit. The simulation time per second of real time simulated using this machine was about 1 day for a simulation with 30 000 particles. The number of particles in the riser depended largely on the drag model and the gas velocity set and ranges between 20 000 and 140 000.

### 2.5 Cluster Detection

**Figure 3:**
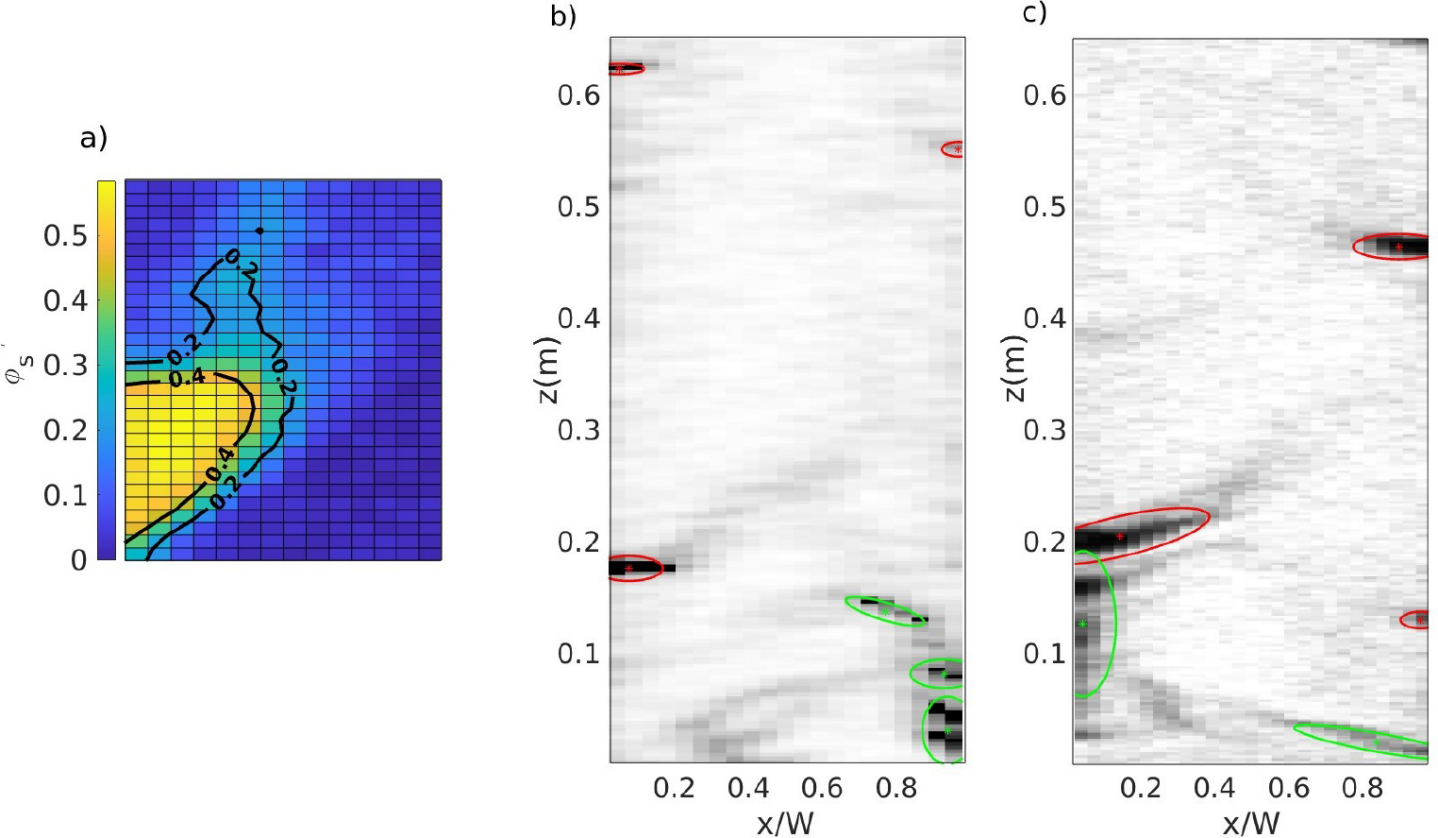
(a) Cluster pixels classified according to the constant thresholds for the wake and core section. (b,c) Final clusters detected for an experimental and numerical frame, respectively. In (b,c) the clusters are labeled as ascending (green) and descending (red) clusters.

Because the general definition of clusters (i.e., regions with a high solids fraction that are 1 or 2 orders of magnitude larger than a particle) is relatively broad, there are multiple clustering detection techniques, e.g. perturbation analysis, image processing, and wavelet transform.^35^ In this study, we implement the best methodology, as proposed by Varas et al.,^30^ where all clusters detected present a core–wake structure defined by two well-defined boundaries of solids volume fraction. One boundary isolates the cluster from the surrounding dilute phase, ε_s_ = 0.20, and the second boundary distinguishes the border between the wake and the core of the cluster, ε_s_ = 0.40.^30^ Although the method will not detect all clusters, the significant clusters responsible for most of the heterogeneity are captured. Because the core–wake structure method allows for setting the thresholds over the full set of frames, the method has proven to have quantitative advantages over methods that depend on flow perturbation or data density distribution, which lead to thresholds varying frame by frame.^30^ This detection method requires a 2D field of the solids fraction in the full riser. In the experiments, this 2D solids fraction can be easily obtained from the high-speed images using digital image analysis (DIA) because of the pseudo 2D nature of the setup. Further details regarding this experimental treatment can be found in Varas et al.^30^Figure 3a presents a visual example of the classification into the core and wake of the cluster. The properties of the obtained clusters are determined using the alphaShape toolbox of MATLAB. Figure 3b,c presents examples of clusters detected for an experimental and numerical frame, respectively.

## 3 Results

### 3.1 Solids Volume Fraction and Solids Flux

**Figure 4:**
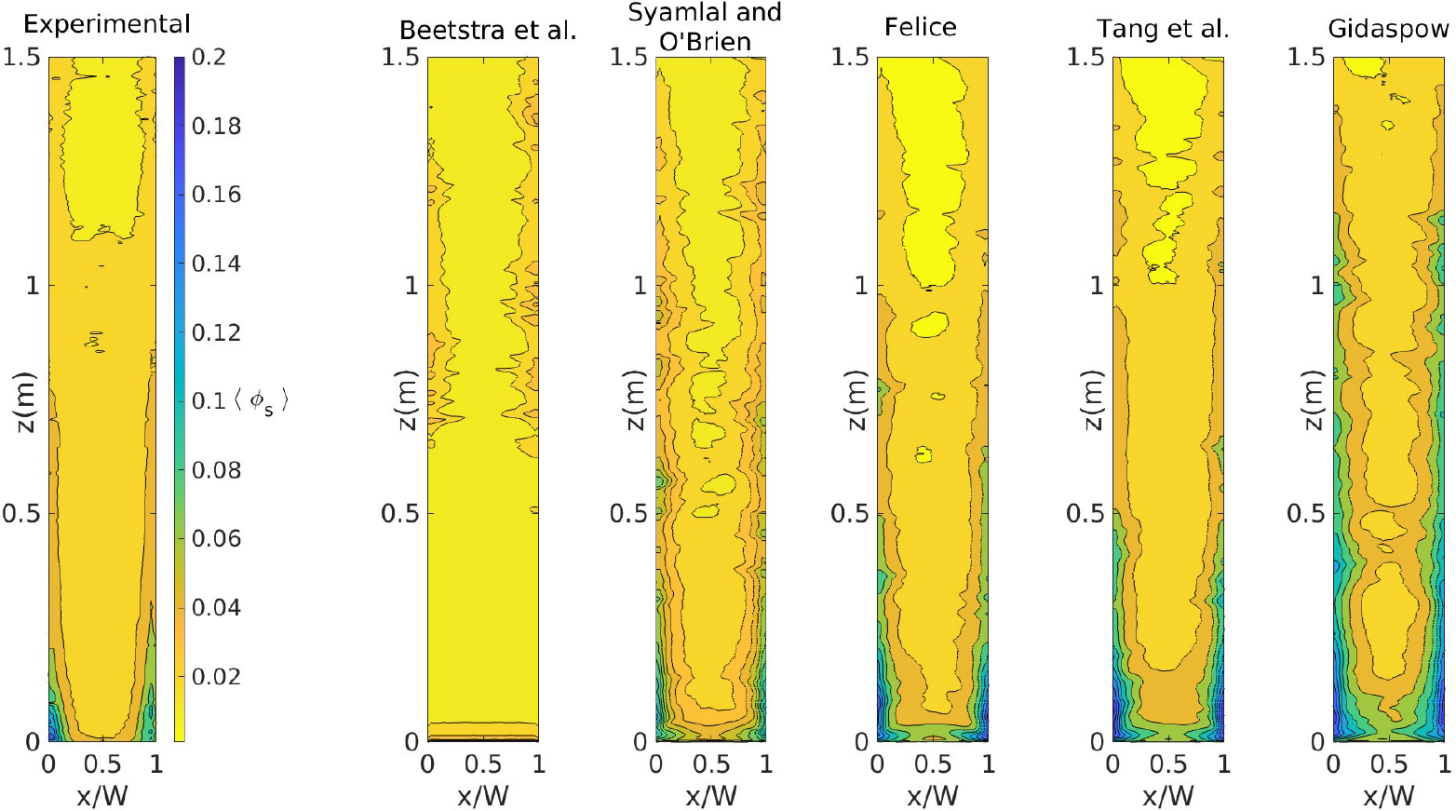
Time-averaged solids volume fraction for *U*_g,in_ = 5.95 m/s.

Figure 4 presents the time-averaged solids volume fraction distribution in the riser with *U*_g,in_ = 5.95 m/s. First of all, both experimental and numerical results show the expected core–annulus behavior. Despite this common feature, there is a large difference in the predictions of the averaged solids volume fraction profile using the different drag force formulations. The simulation results are intentionally placed in increasing order of volume fractions from Beetstra et al. to Gidaspow. Although these models have been the focus for the previous comparison using CFD-DEM,^32^ they clearly under- and overpredict the experimental data, respectively, which indicates the relevance of this study. Results obtained with the Felice and Tang et al. drag formulations present a closer resemblance to the experimental data.

**Figure 5:**
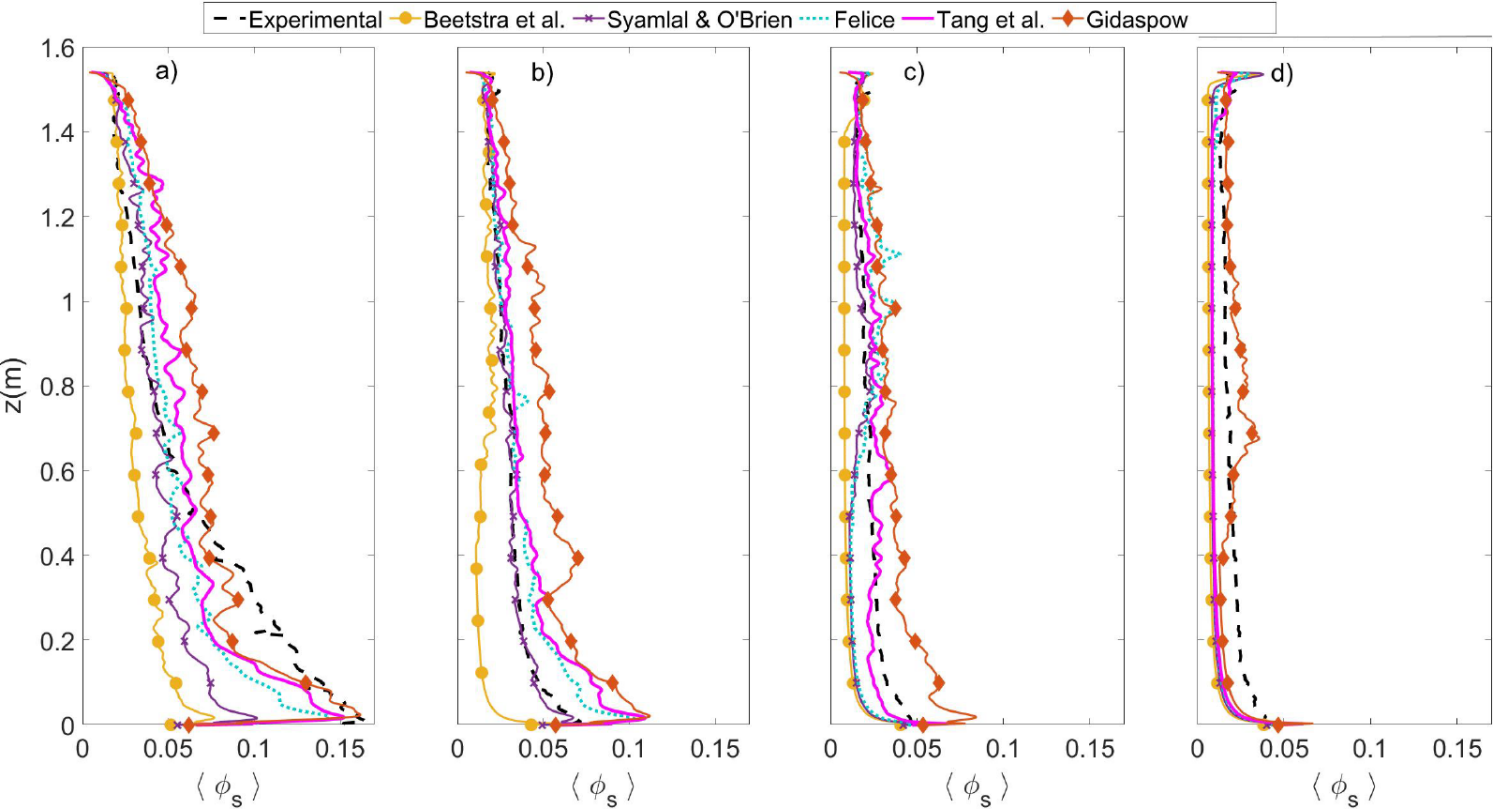
Axial profiles of time- and laterally averaged solids holdup: (a) *U*_g,in_ = 5.55 m/s, (b) *U*_g,in_ = 5.95 m/s, (c) *U*_g,in_ = 6.35 m/s, (d) *U*_g,in_ = 6.74 m/s.

Figure 5 presents the time- and laterally averaged axial profile of the solids volume fraction. Regardless of the gas velocity, the axial profiles show a denser bottom section and a decrease until the dilute top section, as expected. By examining Figure 5a–d from left to right, it is evident that the riser flow becomes more dilute with increasing superficial velocity, especially in the bottom section of the riser. Second, the transition between the solids holdup from the bottom to the top changes from gradual at the lowest velocity (5.55 m/s) to sharp at the highest velocity (6.74 m/s), which results in a near constant value of the solids hold up in the full riser. This sharp profile is associated with a riser operating in a pneumatic transport regime where the particles’ slip velocity reaches a value close to its terminal velocity. Through comparison of the drag formulations, the same order (i.e., from dilute to dense in terms of solids density) is also present for the other gas velocities in the axial profiles of Figure 5. Not only is the correlation of Beetstra et al. always predicting the most dilute riser, it shows the pneumatic behavior even for intermediate gas velocities, as shown in Figure 5b, while the Gidaspow correlation displays the highest solids holdup values along the whole riser for all the superficial gas velocities clearly overestimating the experimental data. The Syamlal and O’Brien correlation is always predicting the second most dilute behavior and, except for *U*_g_ = 5.95 m/s (see Figure 5b), is always under-predicting the solids holdup, especially in the bottom section of the riser. The predictions made with the correlations proposed by Felice and Tang et al. present an intermediate behavior, which both show a close resemblance to the experimental results. Although the experimental profiles in Figure 5 do not give an idea of its uncertainty, Varas et al.^28^ demonstrated that the maximum error in measurements was 18.33%, which is significantly lower than the relevant differences discussed above between predictions and experiments.

**Figure 6:**
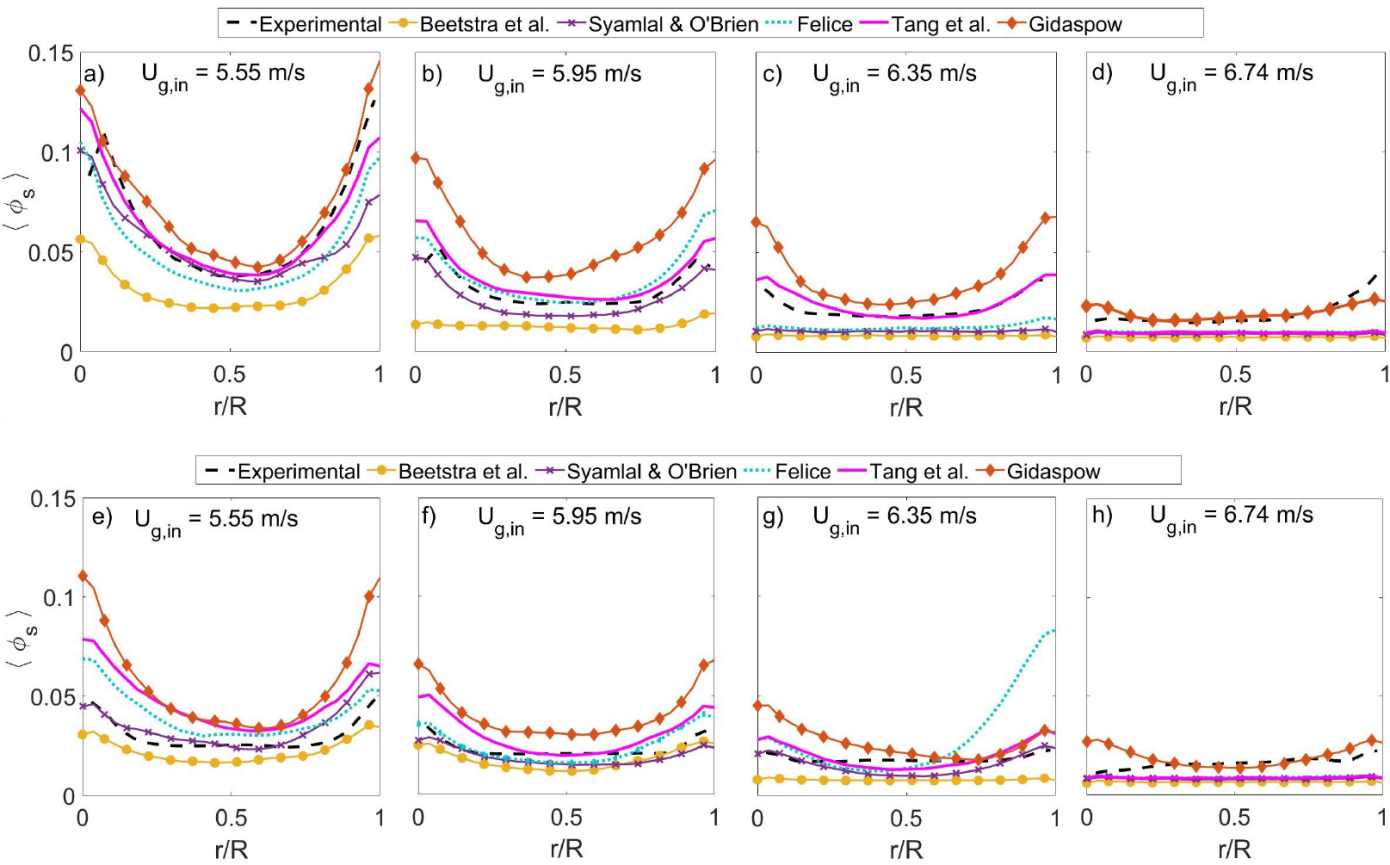
Radial profiles of time-averaged solids holdup as a function of the gas inlet velocity. The profiles in the bottom section of the riser (*z* = 0.5 m) are shown in panels (a–d) for *U*_g,in_ = 5.55, 5.95, 6.35, and 6.74 m/s, respectively. The second row shows the profiles at the top section of the riser (*z* = 1.1 m) in panels (e–h) for *U*_g,in_ = 5.55, 5.95, 6.35, and 6.74 m/s, respectively.

Figure 6 presents the radial profiles of the time-averaged solids holdup for the bottom section (first row) and top section (second row) of the riser for the different gas velocities. In both sections of the riser, an increase in gas velocity tends to flatten the radial solids holdup profiles, which is captured by all the drag formulations. Nevertheless, significant differences are observed when comparing the absolute values. The predictions with the formulations of Tang et al. and Felice are the closest to the experimental values, except at the highest superficial inlet velocity where the Gidaspow model is the only model predicting solids holdup values comparable with the experimental values.

The time-averaged solids flux profile in the riser is computed using eq 7.
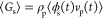
7

**Figure 7:**
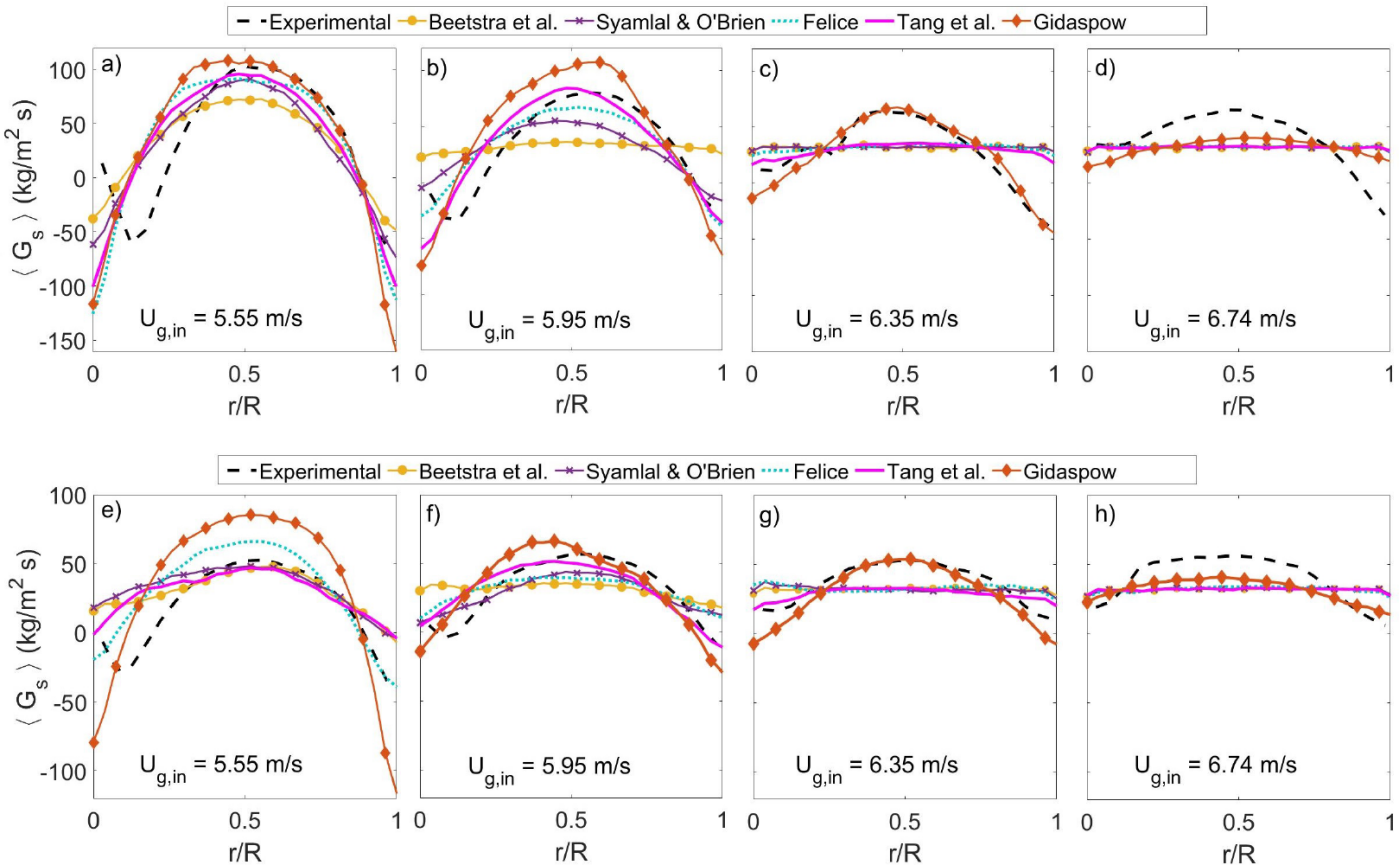
Radial profiles of time-average solids flux as a function of the gas inlet velocity. The profiles in the bottom section of the riser (*z* = 0.5 m) are shown in panels (a–d) for *U*_g,in_ = 5.55, 5.95, 6.35, and 6.74 m/s, respectively. The second row shows the profiles at the top section of the riser (*z* = 1.1 m) in panels (e–h) for *U*_g,in_ = 5.55, 5.95, 6.35, and 6.74 m/s, respectively.

The time-averaged solids flux profiles are presented for both the bottom section (top row) and the top section (bottom row) in Figure 7. The solids flux profiles present the same trend as the solids holdup flattening out as the gas velocity increases. A common feature between the solids holdup profiles in Figure 6 and the corresponding solids fluxes in Figure 7 is the ascending order between the predictions. Surprisingly in Figure 7, an increase in the velocity seems to have a stronger flattening effect on the computed profiles compared with the experimental profiles. This is particularly clear in Figure 7d,h, where none of the five models can predict the radial variation in solids flux properly. As both properties are related, the hypothesis for this difference is that the radial profiles of the particles velocities are flattened more compared with the experiments with increasing superficial gas velocity. This is supported by the large differences in the solids flux, while the solids holdup is well predicted, e.g., the predictions of the Gidaspow model in Figure 6d,h compared with Figure 7d,h. Another example is the prediction using the correlation of Tang et al. in Figure 6c and Figure 7c.

### 3.2 Solids Inventory of the Drag Force Formulations

**Figure 8:**
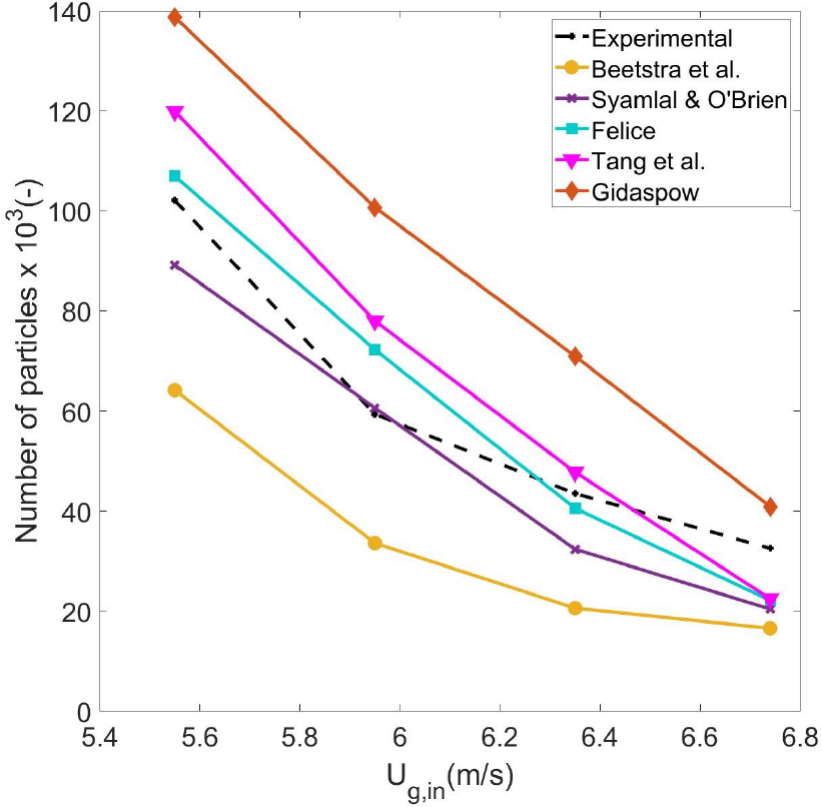
Number of particles inside the riser in pseudo-steady state as a function of the injected gas velocity.

Figure 8 shows the number of particles inside the riser in pseudo-steady state as a function of the superficial gas velocity. The experimental data in Figure 8 was obtained by collecting images of the packed configuration after switching off the air supply when the riser was operating in pseudo-steady state. On the basis of calibration experiments, the number of particles in the bed could be estimated from the particles density in the images. The number of particles in the system show the same order between the correlations regardless of the considered gas velocity. Similar to the results presented in Section 3.1, the Beetstra et al. correlation tends to under-predict the experimental data for the different gas velocities, while the Gidaspow correlation significantly overpredicts the number of particles. These differences decrease at the highest velocity. The correlations of Felice and Tang et al. produce intermediate results, and the number of particles they predict are relatively close to the experimental values except at the highest velocity, which also showed poor predictions for the solids holdup and flux profiles. The Syamlal and O’Brien correlation is also in the intermediate region, but generally predicts a too low number of particles in the system. This order between the models suggests a similar trend in the drag force exerted on the particles at comparable operating conditions because a higher drag force results in more pneumatic transport behavior and, therefore, a lower number of particles.

**Figure 9:**
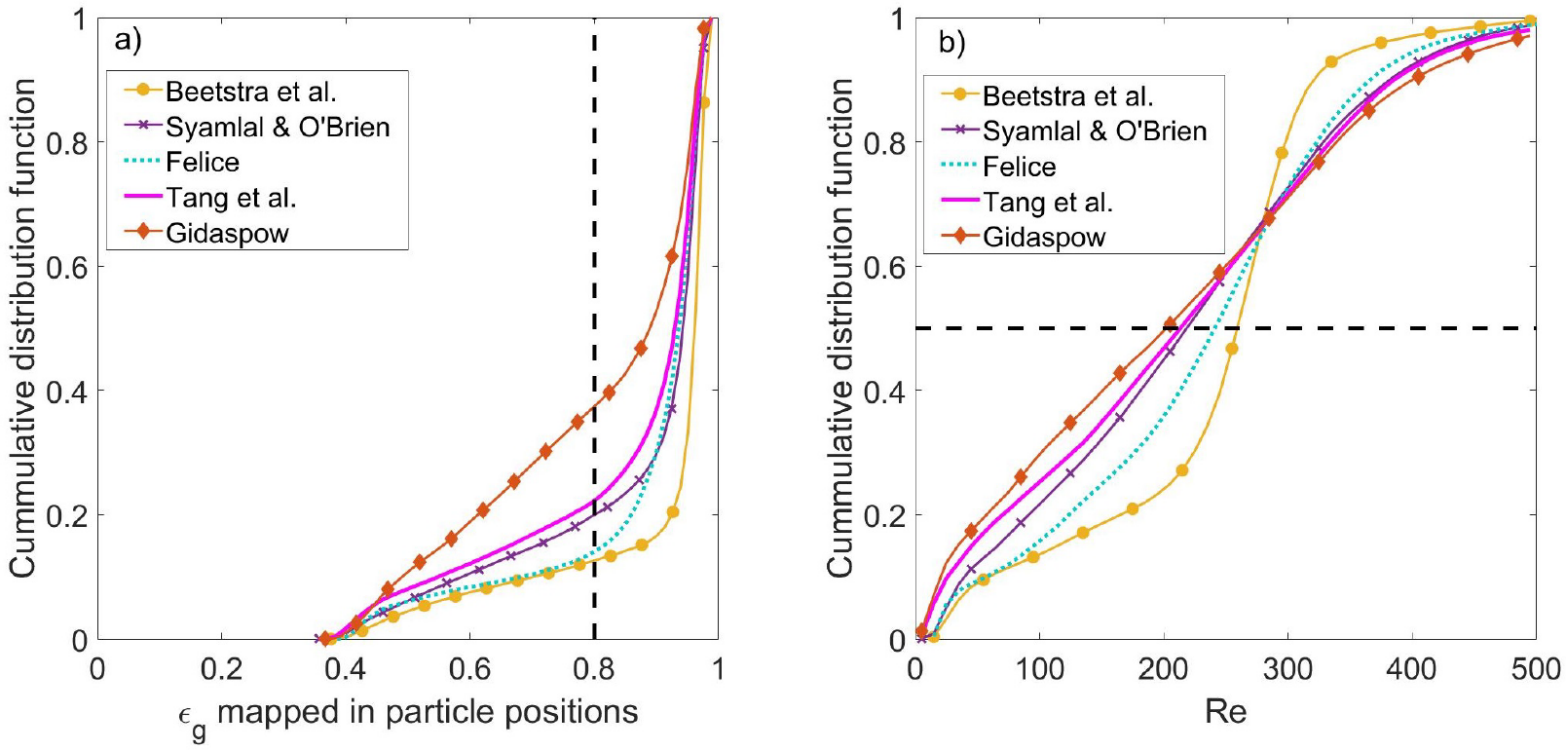
Cumulative distribution function of (a) the porosity mapped at the particle positions and (b) Reynolds number. Profiles are presented for the different drag formulation at *U*_g,in_ = 5.95 m/s.

As discussed in the introduction, the applicability of a drag model is difficult to determine given the wide range of porosity values and Reynolds numbers prevailing inside the riser. The order of the correlations in solids holdup does not naturally follow from the order in the normalized drag force, where the ordering depends on the Reynolds number or solids fraction. To further evaluate the different correlations, the probability density in the Reynolds numbers and solids holdup fractions is presented in Figure 9 at an intermediate gas velocity (*U*_g,in_ = 5.95 m/s). Figure 9a shows that despite the wide range of possible porosity values and independent of the drag formulation, the percentage of particles with a local porosity higher than 0.8 is between 63% and 87%. This suggests that to further understand the relative difference between the models, Figure 1c (and not Figure 1b) is the most representative for the hydrodynamic behavior of the riser. This is confirmed by the behavior obtained with the Gidaspow model. Because of the discontinuity at ε_g_ = 0.8 (see Table 1) for the Gidaspow correlation, there is a clear jump in the predicted drag force at ε_g_ = 0.8 (see Figure 1a). As the model produces a significant overprediction of the solids holdup, the model should operate mainly in the high porosity regime (ε_g_ > 0.8). In addition, the order of the drag models presented in Figure 1c corresponds exactly with the order in their predictions of solid density.

Figure 1c also suggests that the relative differences in the drag force exerted by the different models are more pronounced as the Reynolds number increases. This can explain the significant differences between predictions of the riser given the high Reynolds numbers obtained in the simulations, as shown in Figure 9b. In all models, more than half of the particles experience a Reynolds number higher than 200, and a significant fraction experience Reynolds numbers around 400.

### 3.3 Clustering Behavior

#### 3.3.1 Cluster Frequency and Size

**Figure 10:**
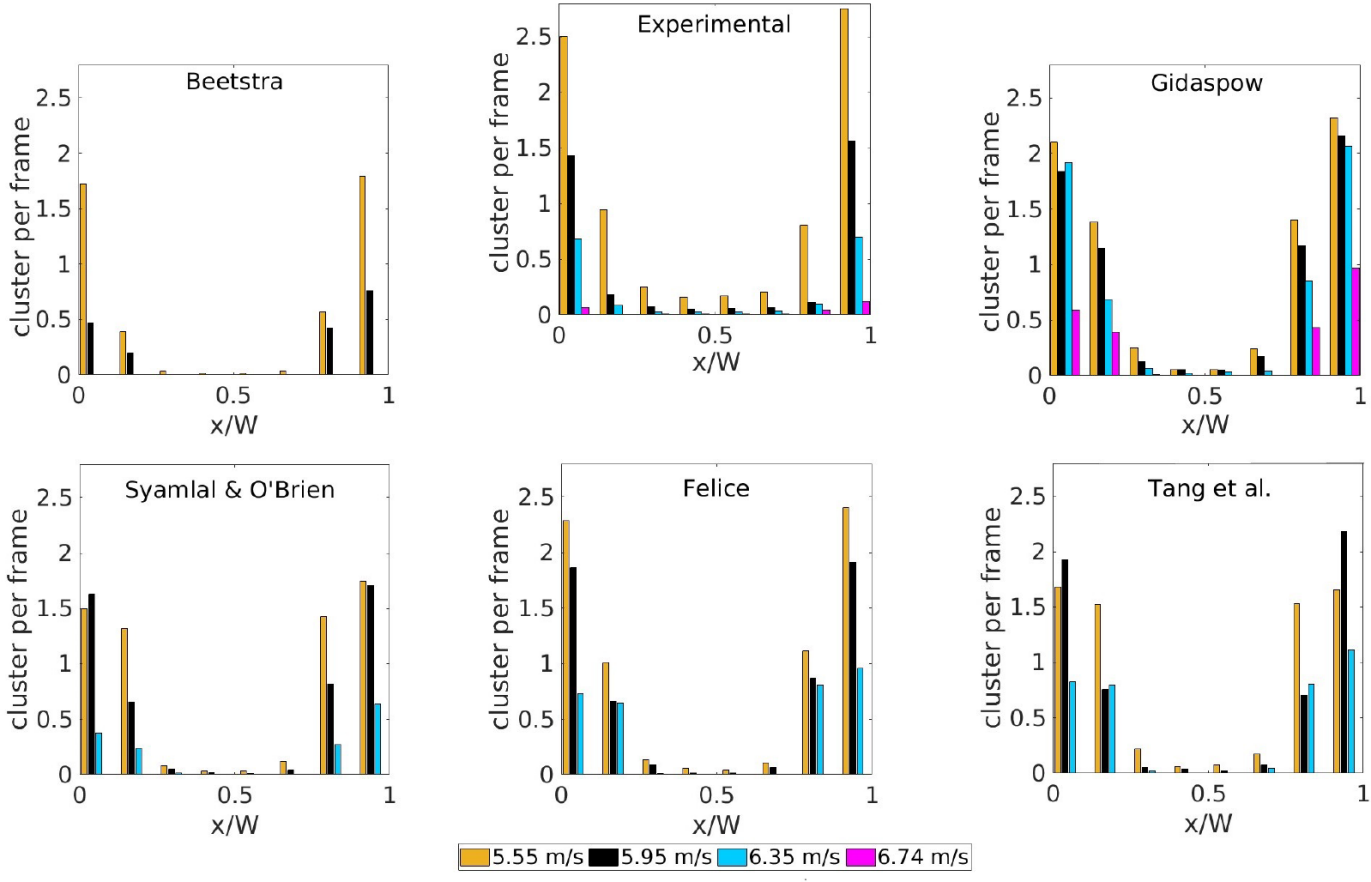
Cluster frequency as a function of the injected gas velocity.

**Figure 11:**
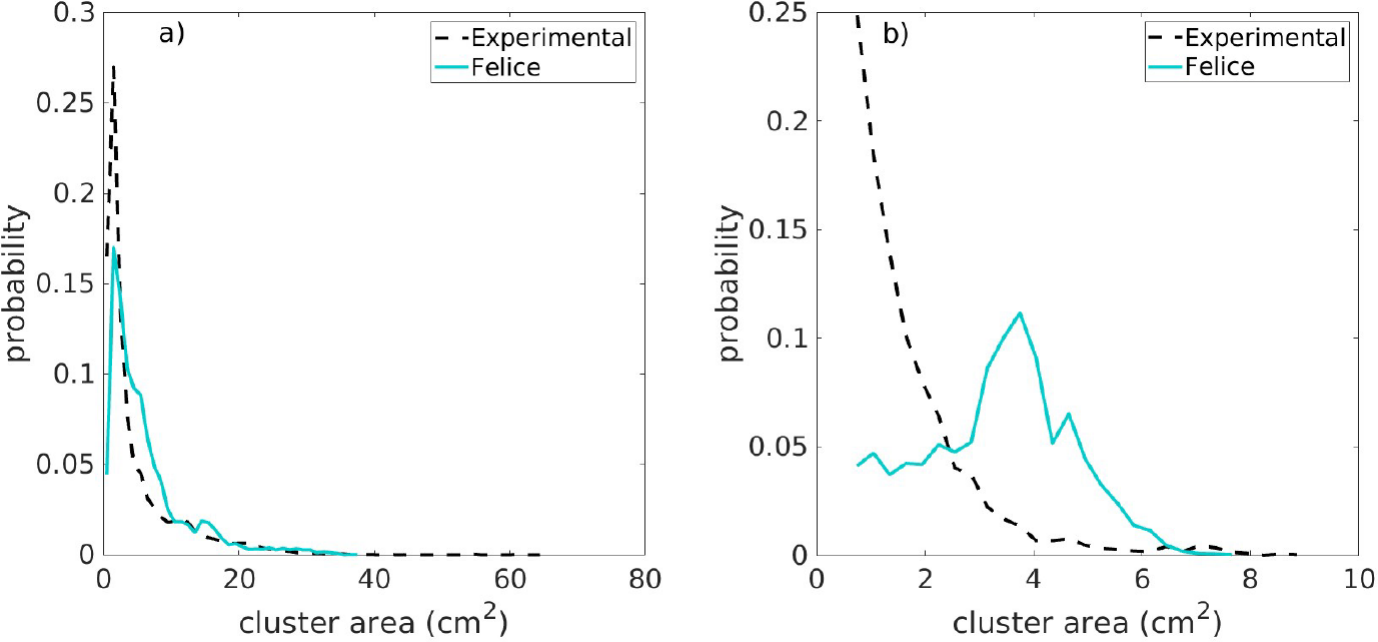
Cluster area probability distribution function: (a) 5.55 and (b) 6.35 m/s.

Figure 10 presents the radial profiles of the cluster frequency, which is defined as the number of clusters detected per frame. As expected, there is a higher density of clusters near the wall, which decreases drastically toward the center of the riser. As the solids content diminishes with increasing gas velocity, the number of clusters decrease to almost zero at the highest velocity. The same order of the results with different correlations is evident in Figure 10; the Beetstra et al. drag closure features almost no cluster formation at gas velocities of 6.35 or 6.74 m/s, while Gidaspow predicts, in general, a higher cluster frequency in comparison with the experimental data. The correlations of Felice and Tang et al. exhibit a similar behavior and are the closest to the experimental distribution. In the experiments, there is a steep decrease in the frequency when moving from the wall to the center regardless of the velocity. However, some of the simulations show a more even distribution of the frequency near the wall (e.g., Felice at *U*_g,in_ = 6.35 m/s or Tang et al. at *U*_g,in_ = 5.95 and 6.35 m/s), which indicates a shift of the centroids of part of the clusters toward the center of the riser. This phenomenon can be explained by the prediction of bigger clusters in these simulations in comparison with the experiments. To explore this hypothesis, an analysis of the size distribution is presented in Figure 11 for a simulation without (Felice at *U*_g,in_ = 5.55 m/s, Figure 11a) and one with shift (Felice at *U*_g,in_ = 6.35 m/s, Figure 11b). In Figure 11a, both the simulation and experimental results show a similar distribution and absolute values, thereby supporting the good correspondence in Figure 10. However, the distribution of the cluster area is shifted to larger sizes for the simulations when a shift of the centroids is observed in Figure 10 (Figure 11b). This confirms the mentioned hypothesis that the shift is caused by a difference in the cluster size.

**Figure 12:**
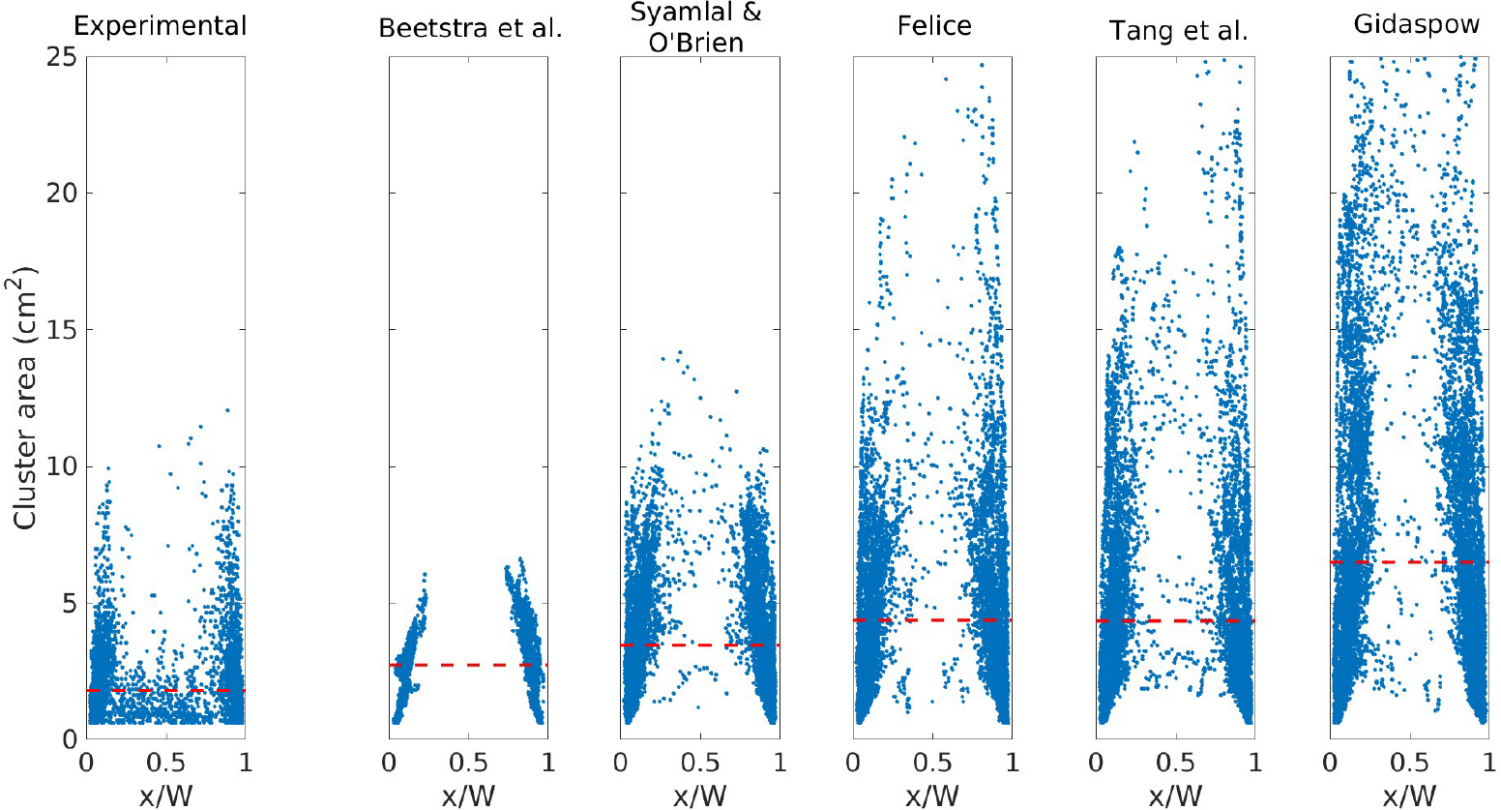
Cluster area versus centroid: *U*_g,in_ = 5.95 m/s. Dotted line represents the average value.

A comparison of the cluster area for the experiments and for the different drag formulations is presented in Figure 12 for *U*_g,in_ = 5.95 m/s. Although the experimental and simulated cluster frequencies are comparable at this velocity (see Figure 10), there are significant differences in the cluster area presented in Figure 12. The average cluster area shows that the different drag formulation could result in clusters that are twice as big, e.g. the cluster area of Beetstra et al. and Gidaspow. This is a consequence of the difference in solids density predicted by the drag formulations. Second, it can be seen that the average cluster area for all the simulations is higher than in the experiment.

#### 3.3.2 Solids Holdup and Velocity

**Figure 13:**
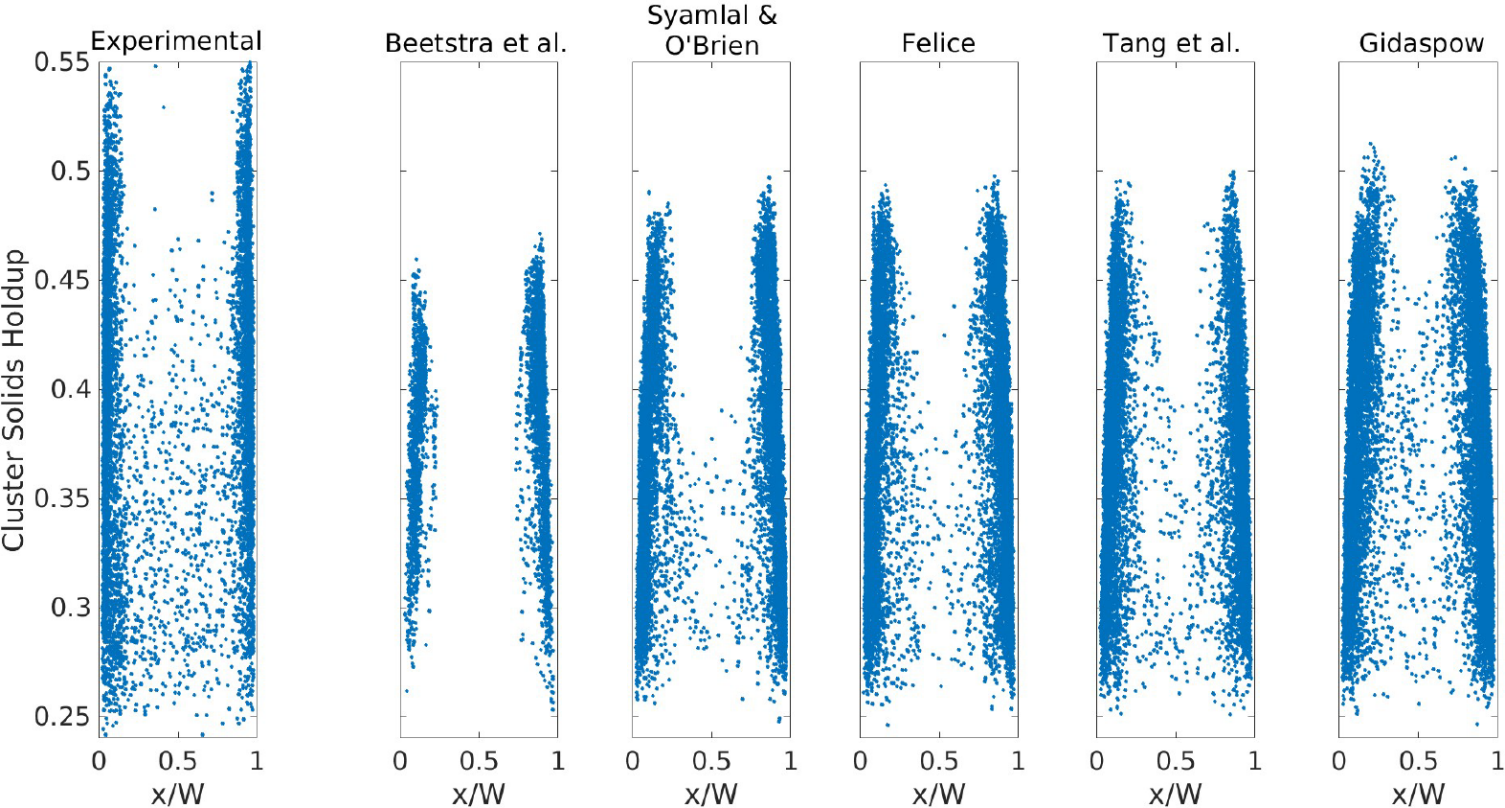
Cluster solids holdup versus centroid: *U*_g,in_ = 5.95 m/s.

The cluster solids holdup was computed as the average solids holdup of the cells defining the cluster, which has a minimum of 0.2 because of the definition of a cluster. In Figure 13, the cluster solids holdup is plotted for both the experimental and simulation results at *U*_g,in_ = 5.95 m/s. The figure clearly shows that the experimental clusters can attain a solids volume fraction of 0.5, which is not reached by any of the drag formulations. This could be related to the larger cluster area predicted in the simulations (see Figure 12), which normally implies clusters with a larger wake region and, thus, a lower average solids holdup. In addition the drag formulation affects the cluster density in the core region of the riser. The correlation of Beetstra et al. produces virtually no clusters in the center of the riser. Figure 13 also shows that a decrease in the drag force (i.e., moving to the right in Figure 13) results in a higher particle density in the core of the riser and, thus, more clusters. Finally, the clusters tend to shift to the center in the simulations with clusters with a higher solids holdup, which can be associated with the larger cluster size, as discussed in Section 3.3.1.

**Figure 14:**
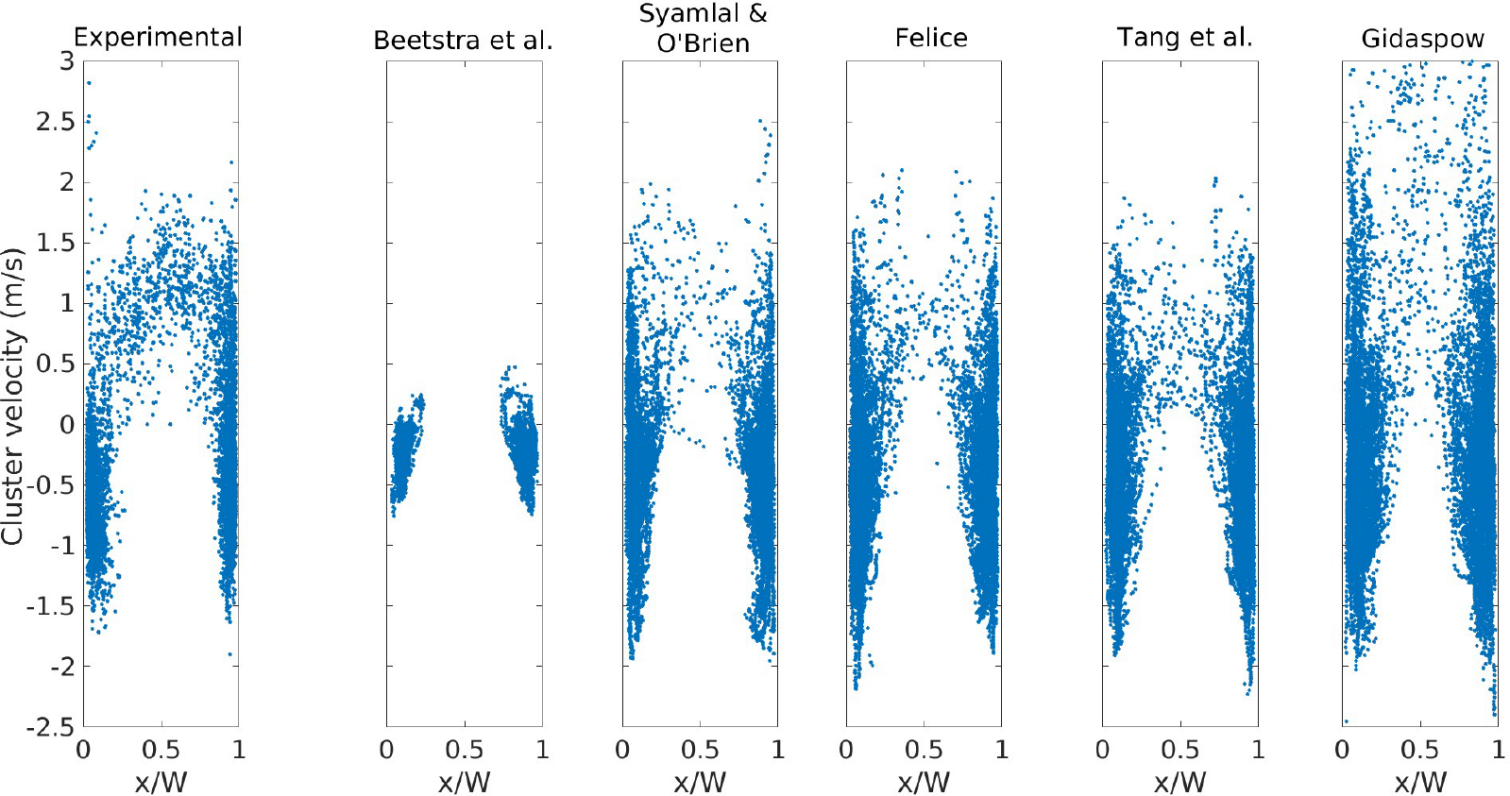
Cluster velocity versus centroid: *U*_g,in_ = 5.95 m/s.

The average axial velocity of the clusters is presented in Figure 14 for the case with *U*_g,in_ = 5.95 m/s. In all plots, the clusters near the wall move downward, which promotes the solids back-mixing in risers. The cluster velocity predicted by the correlation of Beetstra et al. is very low, which is related to the small sizes of the cluster predicted (as shown Figure 12) and the near plug flow behavior of the solids (as shown in Figure 7b,f). The predictions of the averaged cluster velocity using the correlations of Felice and Tang et al. are the closest to the experimental behavior, especially in the core region. The velocities obtained from the Gidaspow model exceed 2.5 m/s, which is rarely observed in the experiments.

## 4 Conclusions

In this work, five different drag force formulations were used in a CFD-DEM simulation of a lab-scale riser for gas velocities between 5.55 and 6.74 m/s. A significant effect of the used drag force formulation was identified in terms of predicted solids density, solids flux, and cluster characteristics (frequency and size). The solids content increases when changing the drag force correlation of Beetstra et al. to Syamlal and O’Brien, Felice, Tang et al., and finally Gidaspow. This ordering can be explained by the ordering of the nondimensional drag force in dilute conditions, which predominantly exists in the riser. In addition, the analysis of the nondimensional drag force also suggests that the differences between the drag formulations become more pronounced at the high Reynolds numbers, which are found in the riser.

All of the drag models have difficulty in predicting the experimental behavior of the riser for the full range of velocities. As the effect of gas velocity on the solids content was not fully captured by any of the correlations, the accuracy of the drag formulations depends on the applied gas velocity. Nevertheless, the correlations proposed by Felice and Tang et al. were close to the experimental data regarding both time-averaged quantities and clustering behavior, except for the highest velocity where pneumatic conveying commences. In this regime, only the Gidaspow drag closure was able to predict axial and radial gradients.

The drag formulations also had a strong impact on the clustering behavior, i.e., the number density of clusters changed by a factor of four, and the sizes of the clusters changed by a factor of two. In the entire range of velocities, the cluster properties were generally only predicted accurately using the drag closures of Felice or Tang et al., except for the highest velocity where the correlation of Gidaspow drag closure predicted a sufficiently high solids inventory to form clusters. However, the Gidaspow model overestimated the frequency and area of the clusters compared with the experiments.

Finally, it is important to mention that the obtained results are only valid in dilute conditions of the riser. In dense operation of the riser, different relative performance of the drag formulations might be obtained.
